# A Smart Surveillance System for Uncooperative Gait Recognition Using Cycle Consistent Generative Adversarial Networks (CCGANs)

**DOI:** 10.1155/2021/3110416

**Published:** 2021-10-13

**Authors:** Wafaa Adnan Alsaggaf, Irfan Mehmood, Enas Fawai Khairullah, Samar Alhuraiji, Maha Farouk S. Sabir, Ahmed S. Alghamdi, Ahmed A. Abd El-Latif

**Affiliations:** ^1^Department of Information Technology, Faculty of Computing and Information Technology King Abdulaziz University, Jeddah 23713, Saudi Arabia; ^2^Faculty of Engineering & Informatics, School of Media, Design and Technology, University of Bradford, Bradford, UK; ^3^Department of Computer Science, Faculty of Computing and Information Technology, King Abdulaziz University, Jeddah, Saudi Arabia; ^4^Department of Information Systems, Faculty of Computing and Information Technology, King Abdulaziz University, Jeddah, Saudi Arabia; ^5^Department of Cybersecurity, Faculty of Computer Science and Engineering, University of Jeddah, Jeddah, Saudi Arabia; ^6^Department of Mathematics and Computer Science, Faculty of Science, Menoufia University, Shibin Al Kawm 32511, Egypt

## Abstract

Surveillance remains an important research area, and it has many applications. Smart surveillance requires a high level of accuracy even when persons are uncooperative. Gait Recognition is the study of recognizing people by the way they walk even when they are unwilling to cooperate. It is another form of a behavioral biometric system in which unique attributes of an individual's gait are analyzed to determine their identity. On the other hand, one of the big limitations of the gait recognition system is uncooperative environments in which both gallery and probe sets are made under different and unknown walking conditions. In order to tackle this problem, we propose a deep learning-based method that is trained on individuals with the normal walking condition, and to deal with an uncooperative environment and recognize the individual with any dynamic walking conditions, a cycle consistent generative adversarial network is used. This method translates a GEI disturbed from different covariate factors to a normal GEI. It works like unsupervised learning, and during its training, a GEI disrupts from different covariate factors of each individual and acts as a source domain while the normal walking conditions of individuals are our target domain to which translation is required. The cycle consistent GANs automatically find an individual pair with the help of the Cycle Loss function and generate the required GEI, which is tested by the CNN model to predict the person ID. The proposed system is evaluated over a publicly available data set named CASIA-B, and it achieved excellent results. Moreover, this system can be implemented in sensitive areas, like banks, seminar halls (events), airports, embassies, shopping malls, police stations, military areas, and other public service areas for security purposes.

## 1. Introduction

Biometric systems employ the human unique characteristics that are either physical or behavioral to determine their identity. All of these characteristics distinguish one individual from the other [1]. Similarly, a Gait Recognition system is also a biometric system that identifies an individual based on the way they walk. It is a relatively new technology that has attracted a large number of researchers in recent years. Previously developed biometric systems, such as face recognition, iris recognition, and fingerprint recognition, used physical attributes of individuals to establish their identity, but gait recognition examines individual's behavior to identify them, making it a behavioral biometric system [2]. The advantages of gait recognition biometric over visual biometrics include that gait recognition is appropriate and effective in recognizing people even when their faces are covered and also when the distance between surveillance cameras and individuals is about 50 meters. On the other hand, iris recognition requires that an individual must be at a distance of 3 cm from the camera, and face recognition requires about 5 meters. Besides this, visual biometrics also requires high-quality images for accurate identification and requires the involvement of the subject during identification. On the other hand, gait recognition is such an admirable biometric system that does not require subject cooperation at the time of inspection from surveillance cameras, and this aspect makes it effective in ensuring security at public safety areas [3].

Different researchers carried out gait recognition in two different experimental settings. One is the cooperative manner, and other is the uncooperative environment. In a cooperative manner, the model is trained and tested on known walking conditions [4]. More specifically, if a model is trained on several individuals who walk normally in a video without carrying any objects or items and then tested on different videos of individuals but with the same walking condition, i.e., normal walk. In this case, the walking condition of the individual being examined is known to the model during validation. On the other hand, in an uncooperative manner, the individual walking condition is kept hidden from the model. The model is trained on a set of individuals who walk with normal style and tested on same individuals but with different walking styles such as a walk with carrying items, such as bags and suitcases in their hands, or with varied clothing conditions such as coats and jackets. These items, or varied walking conditions, are referred to as covariate factors, and they cause individual gait characteristics to be disrupted. Moreover, the second experimental setting is more realistic and very challenging task in computer vision because the performance of the gait recognition model drastically drops when individuals come with unknown and variable covariate conditions. All these are dynamic walking conditions and strongly affect the recognition rate of identifying individuals from their gait. In existing works, when the subjects are considered as cooperative, that is, covariate conditions are known during the training of the system, then the model or system reveals a very superior performance overall. However, there is a drop in outcomes in an experimental scenario when uncooperative environments are considered, and gallery and probe sets are formed under unknown walking conditions.

In this research, we attempt to improve the recognition performance of the deep learning model with the presence of these covariate factors. So in order to improve the results and make the system strong to any dynamic change, we employ cycle consistent generative adversarial networks (CCGANs) [5]. The main objective of this research is to train a model with one walking condition of an individual that is normal walk and validate and test the model with unknown and dynamic walking conditions, i.e., what if a person comes with a bag or a coat and any other dynamic real-time condition. We first utilized a deep CNN model, which is train on a unique gallery set on all individuals with normal walk style. In the second stage, we validated the model with individuals carrying a bag or wearing coats or any other thing. The presence of these covariate factors modifies the gait features of individuals and is different from the features that are extracted by the CNN on a normal walk. So, before passing the tested image directly to trained CNN model, it first goes via CCGANs, which translates a Gait energy image (GEI [6], a more compact gait representation) disrupted with varied covariate factors to a regular walking GEI and recovers the gait features on which CNN is trained to recognize the person. CCGANs are trained in an unsupervised manner, that is, during translation of disrupted GEI to normal GEI, it automatically picks up the right normal GEI from the target domain for disrupted GEI using cycle loss. In addition, currently, different types of GANs are used for different types of tasks. For example, a very basic GAN [7] generates specific artificial images on which it was trained from a random vector, and there is no control over the data that are generated by the model. In our case, we do not want to generate artificial data from a random vector; therefore, basic types of GANs are not applicable. Similarly, conditional GANs [8] are used when we want to convert an image from one domain to an image from a different domain. In this case, we must have paired data, for example, if we want to translate map photographs to aerial photographs, then we must specify that a given instance of the map image is translated to a specific image of aerial photograph during training. In the presented problem, the disrupted probe set needs to be translated into a normal probe set. But the probe set contains images of various individuals, and it would be unjust to indicate at the testing time that a certain GEI of a person walking with bags, such as Person-ID-001, is translated into Person-ID-001 with a normal walk. Because at testing time, we do not know the person's actual label or ID. So, conditional GANs are also not suitable for this problem. However, CCGANs do not have such limitations. So, the main reason for employing CCGANs is that we want to translate the images affected from covariate factors to normal GEIs without revealing the IDs of the persons. CCGANs' unsupervised mechanism is a perfect match for the solution to the challenge at hand because it automatically finds the exact pair for a particular person followed by a translation of the image. The main contributions of this work are as follows:

- Handling the unknown and dynamic walking environment using CCGANs.
- Reconstruction of gait features, which are lost by variable covariate factors.
- CCGANs are not previously used for gait analysis to recover gait features. The experimental findings demonstrate the capability of CCGANs in recovering gait features of individuals for an uncooperative environment and give better performance.
- Comparative analysis of our approach with other methods shows the superiority of the proposed method.

The rest of the article is organized into several sections.  Section 2 presents the related work, Section 3 describes the proposed methodology, and Section 4 presents the results followed by conclusion.

## 2. Literature Review

Currently, gait recognition methods mainly fall into two main approaches: one is model-based approaches [9–11] and the other is appearance-based approaches [12–14]. In model-based approaches, the human body structure is focused to extract gait features with the use of different body parameters, including stride, speed, size of different body parts, and all other static and dynamic parameters. This type of approach is computationally expensive because it requires high-resolution images, even though the gait recognition method is supposed to work with low-quality images and with a minimum amount of light. On the other hand, the appearance-based approaches work on the human silhouettes extracted from different sequences, and mainly, the motion of the human body is focused in this approach. This type of method can work with low-quality images, which are appropriate for real-time public surveillance. The appearance-based approaches are further divided into hand-crafted feature extraction-based methods and deep learning-based methods.

In the context of traditional machine learning methods, Anusha and Jaidhar [12] extract the hand-crafted features from the selective regions of Gait energy image (GEI) [15], which is a popular gait representation using Modified Local Optimal Oriented Pattern (MLOOP) feature descriptor followed by dimensionality reduction algorithm to reduce extracted feature vectors, and then, classification is performed. The proposed approach is validated on the CASIA-B and OU-ISIR B gait data sets, and it works brilliantly in all experiments. Similarly, Lishani et al. [16] also employed the traditional machine learning technique for gait recognition. In this work, Multiscale Local Binary Pattern (MLBP) and Gabor filter bank feature descriptor are used to extract features followed by Spectra Regression Kernel Discriminant Analysis (SRKDA) algorithm for feature selection. In the end, the *K*-nearest neighbor classifier is utilized for classification and achieved the recognition score of 92%. Rokanujjaman et al. [17] introduced a new gait representation termed as frequency-domain gait entropy (EnDFT), from which features are taken from the less affected part of EnDFT, and a distance metric is used to distinguish humans. Similarly, Bashir et al. [13] select the pairwise features using their proposed gait entropy image (GenI) and Adaptive Component and Discriminant Analysis (ACDA). Moreover, Gupta et al. [18] proposed boundary Energy Image (BEI) based gait representation in which contours of all silhouette images of humans are averaged. They use Principal Component Analysis (PCA) for dimensionality reduction and Linear Discriminant Analysis (LDA) for classification and attained encouraging performance.

Currently, deep learning-based methods show exceptional performances in computer vision-related tasks in various domains [19–22]. Similarly, for gait recognition, it is also proved to be an excellent approach in the recognition of individuals from their gait characteristics. Alotaibi and Mahmood [14] proposed a deep specialized convolutional neural network (CNN) with eight layers to classify the subjects. The proposed model is validated on the CASIA-B gait data set with different experimental settings. Hawas et al. [23] proposed the CNNs with the optical flow of GEI to increase the performance of the model. The optical flow excludes the static part of GEI having high pixel intensities and represents only the dynamic part with high intensities of GEI. Similarly, Linda et al. [24] proposed color-mapped contour gait images (CCGIs) and deep CNN for cross-view gait recognition. CCGIs is helpful in discriminating temporal information in human walking sequences. The model is evaluated on CASIA-B gait data set and produces an average accuracy score of 94.65%. Su et al. [25] introduced center ranked loss in their deep neural network to integrate information of all positive and negative samples. Huang et al. [26] proposed the gait recognition model based on keyframes with deep learning techniques. The total frames in a sequence have different contributions towards gait characteristics. For this, they proposed an extraction module that extracts the keyframes from a given gait sequence and results in the extraction of highly distinctive features. Yao et al. [27] proposed a skeleton gait energy image (SGEI) using multibranch CNNs, in which one branch is responsible for predicting confidence maps, and the second branch is used to predict the affinity fields. After that, the results and features of the image from both branches are concatenated to perform gait recognition. Ling et al. [28] employed the attention mechanism-based approach in gait recognition to emphasize discriminating regions. They validate their approach on OU-ISIR TREADMILL data set B to handle the problem of covariate conditions. Moreover, the use of transfer learning is also employed to gait recognition in which Wu et al. [29] proposed the DenseNet-based transfer learning method. The spatial information of gait from GEI is given as an input to DenseNet to extract features of each subject, and finally, the KNN is used to classify individuals respectively.

Furthermore, in some recent research studies, the study of human behavior in different domains is also exploited. In the context of activity recognition, Shu et al. [30] address the problem of group activity recognition in the multiple-person scene. They proposed the Graph LSTM-in-LSTM (GLIL) based framework, which simultaneously models both individual- and group-level activities. Similarly, Tang et al. [31] employed the motion characteristics of humans to solve the problem of group activity recognition. Relevant motions of individuals are captured by the constraint of Context Coherence (STCC) and a Global Context Coherence (GCC). Kabir et al. [32] recognized the activity of humans employing state-space linear modeling. A coefficient matrix is used to define the association between states and inputs. Furthermore, the most advanced research by Shu et al. [33] aimed to predict future motions of individuals based on currently observed motions. Moreover, the limitations of a different algorithm for activity recognition are also well researched. Choe et al. [34] showed the high operation complexity of the KNN algorithm for activity recognition and proposed a method to reduce this complexity. Besides the gait recognition in the field of biometrics, there also exists some other possible scenarios and application areas, such as Big Data and other IoT systems, in which similar kinds of work can be applied. Content analysis of videos is also used in other fields. For example, Song et al. [35] analyzed and reviewed the latest methods on the content analysis of the videos for action recognition. Instead of action recognition, the behaviors of humans are also used for the gesture, speech, and wrist-activity recognition. For example, Jo et al. [36] proposed the novel method for Hidden Markov Model (HMM) based speech recognition. Their approach is based on a modified version of the Viterbi scoring method. In order to determine an optimal matching model, Viterbi scoring plays a remarkable role in speech recognition systems based on HMM. A dynamic recognition system based on human gestures is introduced by Chen et al. [37], which are applicable in IPTV remote control. A hardware accelerator is also designed for object detection of real-time moving objects. These surveillance systems captured the videos from several cameras, and these videos often suffer from various noises. Different researchers also proposed different techniques to overcome the noise problem. For example, Niu et al. [38] proposed an approach to remove blurriness in images of surveillance systems videos during a raining environment. Wang et al. [39] proposed a novel method of noise processing for underwater targets. This approach is further integrated with CNNs to identify the underwater target.

Currently, the advancements in computer vision-based algorithms and techniques are increasing rapidly and adopted for many daily life use cases. One of the most advanced algorithm is generative adversarial networks [7]. They are generative models based on deep learning methodologies and are categorized into supervised and unsupervised models. Cycle consistent generative adversarial network (CCGAN) is one of the types of unsupervised generative models, which performs image translation in unsupervised manner [5]. It is used in most of the computer vision-related domains. Recently, Kearney et al. [40] involved the attention mechanism in CCGANs to perform image translation of MRI to CT scans. Similarly, Armanious et al. [41] translated the Positron Emission-computed Tomography (PET) images to CT scan images using CCGANs along with nonadversarial cycle losses. On the other hand, when it comes to gait recognition, Yu et al. [42] proposed a framework, namely, GaitGAN to select invariant features of an individual's gait to reduce the effect of covariate factors. However, the presented approach using GANs model is trained in the supervised manner in which the source and target GEIs are already known. Similarly, for view-specific gait recognition, He et al. [43] employed the multitask GANs to directly change view specific attributes in the latent space. Furthermore, the alpha-blending GANs are employed by Li et al. [44] to translate the GEIs affected by carried objects to GEIs without carried objects. It is reasonable to draw a conclusion that analysis of human behavior for different kinds of tasks, such as person identification for the biometric systems, speech recognition, gesture recognition, and action recognition, are active research areas in the current era.

## 3. Proposed Methodology

**Figure 1:**
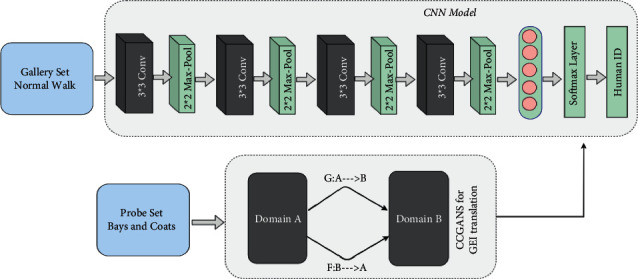
The proposed framework for gait recognition in uncooperative environment.

The proposed methodology of the gait recognition system is presented in Figure 1. As shown in Figure 1, we first train the CNN model on a gallery set, which is composed of individual's GEIs during a normal walk. Later, we used the CCGAN model to convert the image disrupts from covariate factors to regular GEIs and make recognition possible and accurate in the uncooperative setting of experimentation.

### 3.1. Input Data

The popular gait representation, namely, gait energy image (GEI) is used as an input to the proposed approach [6]. It can be computed by first extracting the frames of a sequence of an individual followed by computation of silhouettes using background subtraction from each frame. Then, at the last, all the silhouette images of one sequence of a particular individual are averaged and aligned to form a gait energy image representation. Equation (1) shows the mathematical formulation of GEI:

(1) $$\begin{array}{c} \text{GEI}=G\left(x,y\right)=\frac{1}{T}\sum_{t=1}^{T}I\left(x,y,t\right). \end{array}$$

**Figure 2:**
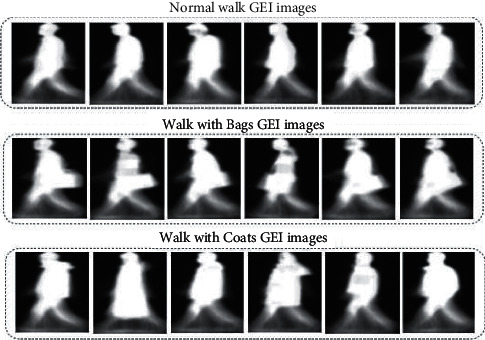
Examples of CASIA-B gait data set in which the first row corresponds to GEIs with a normal walk, the second rows correspond to subjects carrying a bag, and the third row corresponds to subjects wearing coats.

In equation (1), the *G*(*x*, *y*) represents the resulting GEI, where *I*(*x*, *y*, *t*) denotes the silhouette image with frame number *t* and coordinates *x*, *y*, while a total number of frames are represented by *T*. The main advantage of this gait representation is less storage space and computational time. The silhouette images are prone to noise, and processing each silhouette frame is too costly; therefore, GEIs are the most effective gait representation. Furthermore, the GEIs are also very sensitive to different covariate factors, which strongly affect the shape of the GEI. Some examples of gait energy images of the CASIA-B data set with different walking conditions are shown in Figure 2.

### 3.2. CNN Architecture

After extracting the GEIs of all subjects, we resized and rescaled them to 240 × 240 × 1 image dimension and give them as an input to CNN architecture. We have used the same CNN architecture proposed by Bukhari et al. [4]. In this CNN architecture, there are four convolution blocks. Each block is composed of 3 × 3 convolution layers, followed by 2 × 2 max-pooling layers to reduce the spatial dimensions of the input data and select the maximum value from the input region as per the following equation:

(2) $$\begin{array}{c} y_{k.w}^{i}=\underset{0\leq a,b\leq p}{\max}\left(xi_{k\times p+a,w\times p+b}\right). \end{array}$$

In the above equation, a neuron or unit *y*_*k*.*w*_^*i*^ on down sampling layer is present on a particular position (*k*, *w*) in *i*^th^ output map. On the region *p* × *p*, a maximum value is chosen and assigned to *y*_*k*.*w*_^*i*^ neuron in the *i*^th^ input map *x*_*i*_. Subsequently, the activation function used after each convolution layer is Leaky ReLu to achieve nonlinearity. It is defined in the following equation:

(3) $$\begin{array}{c} f\left(x\right)=\left\{\begin{array}{cc} x & \text{if }x>0\\ 0.01x & \text{otherwise} \end{array}\right\}. \end{array}$$

In the above equation, *x* is a feature map resulted from the convolution layer. If the values in *x* are greater than zero, they are preserved; otherwise, they are multiplied by a small value of 0.01. This activation function is an improved form of the ReLu activation. In the case of ReLu activation function, the gradient is 0 for all input values less than zero, deactivating the neurons in that region and perhaps causing the dying ReLu problem. On the other hand, Leaky ReLu overcomes this problem. In the case of Leaky ReLu, instead of converting the gradient 0 for all negative values, it returns a small number multiplied by 0.01 times *x*. This small value shows the influence of negative values in feature maps *x*. The higher the value, the lower the influence. As a consequence, it also produces outputs for negative values. Furthermore, all the convolutions are not padded. The feature maps, which are the output of the convolutional layers, can be computed by the following equation:

(4) $$\begin{array}{c} X=b_{i}\sum_{k=1}^{n}W_{i,j}*Y. \end{array}$$

In the above equation, *X* is the input to the layer and *Y* is the output of layer in which *∗* denotes the operation of convolution, where *b*_*i*_ is bias term and *W*_*ij*_(*i*, *j* ∈ *N*) is weight optimized by weight optimizer algorithm, and size of input data is denoted by *n*. Moreover, all the convolution blocks in our proposed architecture are responsible for extracting the semantic information from the GEI and make a feature set that discriminates each individual based on their gait. At the last, two fully connected layers are used. The number of neurons in the last layer is equal to a number of class labels or individuals present in the data set followed by the “softmax” activation function, which returns the probabilities of every class.

The architecture is trained on 124 individuals present in the CASIA-B gait data set with normal walking conditions. In addition, the epochs are set to 30 with weight optimizer Adam along with a learning rate of 0.0001, respectively.

### 3.3. Cycle Consistent Generative Adversarial Networks (CCGANs) for Reconstruction of Gait Features

#### 3.3.1. Problem Formulation

To satisfy the contribution of the presented work, we have employed the cycle consistent generative adversarial network (CCGANs). The main objective of this study is to handle the unknown and dynamic walking conditions. More specifically, we seek to reconstruct the gait features, which are destroyed by the presence of covariate factors. So, after compiling the input data in the form of GEIs for each subject, we created the CNN model. This CNN model is trained on normal walking sequences (videos) of all subjects, that is, 124. Now, if one of those 124 individuals comes in front of a surveillance camera while carrying a bag for identification, then the system performs poorly due to the occlusion of the bags, which disrupts the gait features. Because when a bag is placed in front of certain areas of the body, they become hidden, and hence, important information regarding the gait style of an individual is also lost. The same is the case with other carrying items and wearing conditions. Therefore, it is difficult to identify the person. We may also consider it as how to recognize the individuals from their faces if faces are covered by masks because masks are occlusions that hide the features of the face. So, a cycle consistent GAN model is deployed to recover/reconstruct the original image or gait features that are affected by these covariate factors. This model was proposed by Zhu et al. for image-to-image translation tasks when the source and target images are not paired [5]. The advantage of this model is that it carries out training without paired images. In our scenario, we reconstruct the GEIs of bags and coats of each individual to a corresponding normal GEI so that features are recovered and human recognition is possible and CNN is able to predict the subject ID with more accurate results. Furthermore, CCGANs efficiently handle dynamic covariate conditions of bags and coats that are unknown to the model; that is, we train the CNN model on individuals with normal walk styles and validate it on individuals' walks while carrying bags and wearing coats. Furthermore, Table 1 shows the symbol to describe all used notations or keywords.

#### 3.3.2. Architecture and Design Mechanism of CCGANs

The model architecture of Cycle Consistent GANs is composed of two generator models and two discriminator models. The generator models are responsible for generating the required images, and discriminator models are used to discriminate between the real and fake images generated artificially. Our source domain contains GEIs of bags and coats (Domain A), whereas the target domain contains the normal walk GEIs of individuals (Domain B). The input of the first generator (generator A) is the images from Domain B, that is, GEIs with a normal walk, and the output is the translated images to Domain A, which is bags and coats GEIs. Similarly, the input of the second generator (generator B) is the images from Domain A, and the output is translated images from Domain B. Each generator model is associated with its corresponding discriminator model. The discriminator A takes input images of bags and coats, which are domain A images along with the artificially generated images from generator A and predicts that whether the images are real or fake. Similarly, the second discriminator B takes normal real GEIs and artificially generated normal GEIs from generator B and then predicts that whether they are real or fake. The adversarial zero-sum loss is used to train both generator and discriminator models. The main objective of the generator model is to best fool the discriminator model with the help of generated images, and the discriminators models are learned to predict and detect the fake and real images of both domains. Furthermore, the CCGAN models use the term cycle because a cycle is created in the whole process. The input of generator B is the images from Domain A, that is, bags and coats, and the output of generator B is the normal GEIs, which are our required translation. This output is the input of the generator A to generate images of domain A and hence the cycle is created. Similarly, the identical procedure is followed with generator A.

The architectural configurations of the discriminator and generator model are the same as described in the previous literature [5]. The discriminator is simply a CNN that performs image classification and predicts that the image is real or fake. A convolution operation, followed by instance normalization instead of batch normalization is used in the discriminator model of the CCGANs. The activation function used in the whole discriminator model is Leaky ReLu. All the convolutions are padded convolution with a kernel size of 4*∗*4. Furthermore, the hyperparameters of the discriminator model include loss function, which is Mean Squared Loss (MSE), and Adam optimizer used for optimizing weights with a learning rate of 0.0002. The formula of computing MSE loss during GEI translation is given by the following equation:

(5) $$\begin{array}{c} \text{MSE}=\frac{1}{n\;}\cdot \sum_{i=1}^{n}{\left(y_{i}-{\hat{y}}_{i}\right)}^{2}, \end{array}$$

where *y*_*i*_ and ${\hat{y}}_{i}$ are the actual and predicted class labels of the model and *n* is the total number of classes. On other hand, the architecture of the generator model is based on an encoder-decoder structure. The generator model first downsamples the image to get the context of the image up to the bottleneck layer and then encodes this context with the help of ResNet layers that uses the skip connections followed by upsampling layers to decode the context to the required output image.

#### 3.3.3. Training Mechanism of CCGANs

The training of discriminator models consists of real and artificially generated images while the generator models are trained with the help of their discriminator models. Generator models use the adversarial loss and update and minimize it from the prediction of the discriminator as “real” for generated images. This encourages generator models to generate images that are closer to our required target domain images, i.e., normal GEIs. Moreover, the other loss functions that are used by the generator model include identity loss, forward loss, and backward loss. For the adversarial loss, consider the mapping function for bags/coats to normal GEI translation as *G* : *A*⟶*B* and discriminator B of domain *D*_*B*_, i.e., normal GEIs, then the objective is expressed in the following equation:

(6) $$\begin{array}{c} L_{\text{GAN}}\left(G,D_{B},A,B\right)=E_{b∼p\;da\;ta\left(b\right)}\left[\log\;D_{B}\left(b\right)\right]+E_{a∼p\;da\;ta\left(a\right)}\left[\log\left(1-D_{B}\left(G\left(a\right)\right)\right)\right], \end{array}$$

where *G* is a generator that learns to generate the required images *G*(*a*) that look closer to images of domain B, i.e., images with recovered gait features, while the objective of the discriminator *D*_*B*_ is to distinguish the generated images *G*(*a*) and real images *b*. *G* is attempting to minimize this objective, whereas adversary *D* is seeking to maximize it.

(7) $$\begin{array}{c} \min G\max D_{B}L_{\text{GAN}}\left(G,D_{B},A,B\right). \end{array}$$

Similarly, for mapping function *F* : *B*⟶*A*, the similar adversarial loss is introduced along with discriminator *D*_*A*_ and computed by the following equation:

(8) $$\begin{array}{c} \min F\max D_{A}L_{\text{GAN}}\left(F,D_{A},B,A\right). \end{array}$$

Moreover, the forward and backward cycle consistency are also computed. Consider an image *a* from the domain *A* for which the reconstruction of gait features is needed. So, for the forward cycle consistency, the whole cycle in CCGAN image translation can bring an image *a* to its original form, i.e. ,  *a*⟶*G*(*a*)⟶*F*(*G*(*a*)) ≈ *a*. Similarly, for backward cycle consistency, we have instance *b* from the domain *B*, and *G* and F should also satisfy the equation of backward cycle consistency: *b*⟶*F*(*b*)⟶*G*(*F*(*b*)) ≈ *b*. So, all the mechanism of cycle consistency loss is given in the following equation:

(9) $$\begin{array}{c} L_{\text{cyc}}\left(G,\;F\right)=E_{a∼p\;da\;ta\left(a\right)}\left[{\left\|F\left(G\left(a\right)\right)-a\right\|}_{1}\right]+E_{b∼p\;da\;ta\left(b\right)}\left[{\left\|F\left(G\left(b\right)\right)-b\right\|}_{1}\right]. \end{array}$$

Furthermore, the full objective is given by the following equation:

(10) $$\begin{array}{c} L\left(G,F,D_{A},D_{B}\right)=L_{\text{GAN}}\left(G,D_{B},A,B\right)+L_{\text{GAN}}\left(F,D_{A},B,A\right)+\lambda L_{\text{cyc}}\left(G,F\right). \end{array}$$

In the above equation, the relative importance of two objectives can be controlled by *λ*. The main aim is to solve the objective given in equation (10):

(11) $$\begin{array}{c} G^{*},F^{*}=\underset{G,F,D_{a},D_{b}}{\arg\min\max}L\left(G,F,D_{A},D_{B}\right). \end{array}$$

It is observed that this model can be viewed as training of two “autoencoders” [45]. One auto encoder *F*∘*G* : *X*⟶*X* learned jointly with the second autoencoder *G*∘*F* : *Y*⟶*Y*. However, both autoencoders have their internal structures, and an intermediate representation is used to map an image to itself that is a translation of image from one domain to another domain. These scenarios can be observed as a special case in the work of “adversarial autoencoders” [46], in which the bottleneck layer of an autoencoder is trained with the help of adversarial loss to generate any arbitrary target. In our case, the domain *B*, which contains normal GEIs of different subjects, is the target distribution for the autoencoder *A*⟶*A*.

## 4. Experimental Setup and Results

### 4.1. Data Set

In our proposed work, we have used a popular data set for gait recognition named CASIA Gait data set provided by the Chinese Academy of Sciences (CASIA) [47]. It is composed of three main parts named CASIA A, B, and C. For this research, CASIA B is considered. CASIA B is a large multiview gait data set with viewpoints starting from 0 to 180 degrees. For each viewing angle, it consists of data of 124 subjects with each subject having a total of ten sequences available. Of ten sequences, six sequences are normal walk sequences [nm-01 to nm-06], two sequences in which subjects are walking with a bag [bg-01, bg-02], and two sequences in which clothing conditions are focused and subjects walk with wearing a coat [cl-01, cl-02].

### 4.2. Evaluation Metrics

To evaluate the proposed model, the performance measure includes the accuracy, F1score, precision, and recall are considered [48]. The detail of each measure is given below:

#### 4.2.1. Accuracy

The total number of true predictions by the underlying model from overall predictions is measured by accuracy, and it is defined in the following equation:

(12) $$\begin{array}{c} \text{Accuracy}=\frac{TP+TN}{TP+TN+FP+FN}. \end{array}$$

#### 4.2.2. Precision

Precision measures the total number of positive class predictions by the model that are in fact positive class predictions, and it is mathematically computed by the following equation:

(13) $$\begin{array}{c} \text{Precision}=\frac{TP}{TP+FP}. \end{array}$$

#### 4.2.3. Recall

Recall measures the total number of positive class predictions by the model from all positive cases, and it is mathematically computed by the folllowing equation:

(14) $$\begin{array}{c} \text{Recall}=\frac{TP}{TP+FN}. \end{array}$$

#### 4.2.4. F1 Score

The both precision and recall metrics are merged in F1 score to measure the entire performance of the model. Mathematically, it is described in the following equation:

(15) $$\begin{array}{c} F1=2\cdot \frac{\text{Precision}\cdot \text{Recall}}{\text{Precision}+\text{Recall}}. \end{array}$$

### 4.3. Results and Discussion

All the simulations with python implementation are run on Google Colab with a single 12 GB NVIDIA Tesla K80 GPU. It is necessary to mention here that in our whole experimental setup, we handle the unknown and dynamic walking conditions; that is, our gallery set consists of all 124 individuals with a normal walking condition with each individual having four sequences [nm-01-nm-04] as used by other researchers [13, 29, 49]. So, our CNN model is trained with this gallery set having 124 individuals. After this, the trained CNN model weights are saved. We created two probe sets for model evaluation, one with clothing condition (coats) and the other with carrying condition (bags) of all 124 individuals. When we input these probe sets directly to our trained CNN model, then the results are poor. The CNN model fails to recognize the same 124 individuals on which it was trained because at this time, all 124 individuals come with an unknown walking condition. They wear jackets and carry bags; thus, these covariate factors alter the gait characteristics of all 124 people, causing the system to fail to detect them. As shown in Table 2, the accuracy of the trained CNN model is only 38% and 24.19% on probe set bags and coats. So as a solution, we recover the features set by employing cycle consistent generative adversarial networks (CCGANs). It converts the data of all 124 persons with bags and jackets to normal walk images and removes all covariate factors. At the time of training of CCGANs, the source and their corresponding target images are not already known; that is, CCGANs automatically find the normal GEI for bag GEI of a particular individual with the help of Cycle Loss. CCGANs eliminate all covariate factors that make recognition difficult. Afterward, the translated data are fed into the CNN in order for the subject to be recognized. Table 2 shows the results of directly inputting covariate data to the CNN and the results of CNN + CCGANs. It is evident from the table that the accuracy of our proposed approach improves with CCGANs due to the reconstruction of gait features. On probe set with bags, we have achieved 79% accuracy, and with coats, 27.61% results are improved. Similarly, the accuracy, recall, and F1 score for bags are 0.72, 0.79, and 0.79, respectively, whereas on coats, they are 0.38, 0.51, and 0.41.

**Figure 3:**
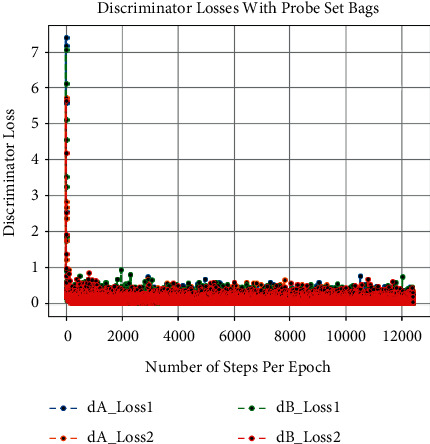
Graphs of different loss functions on both probe sets.

**Figure 4:**
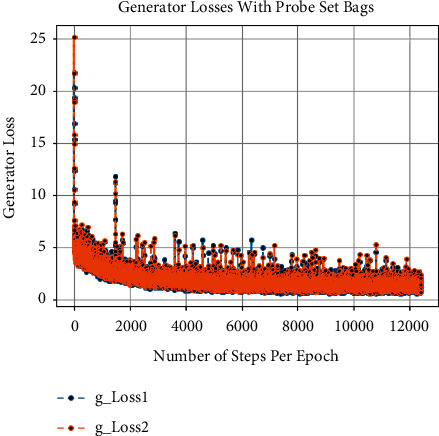
Loss of discriminators on number of steps per epoch with bags.

**Figure 5:**
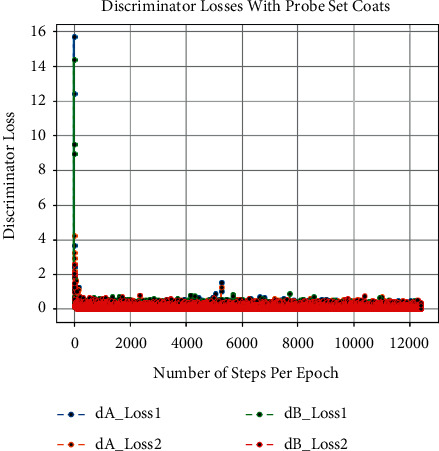
Loss of generators on number of steps per epoch with bags.

**Figure 6:**
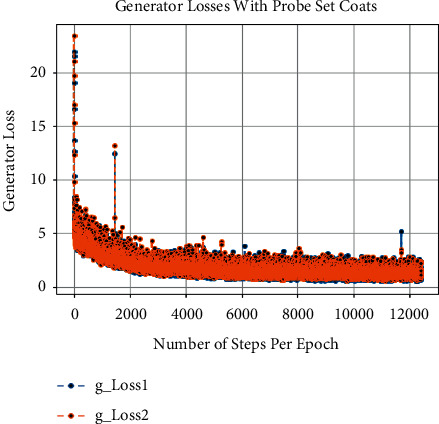
Loss of discriminators on number of steps per epoch with coats.

**Figure 7:**
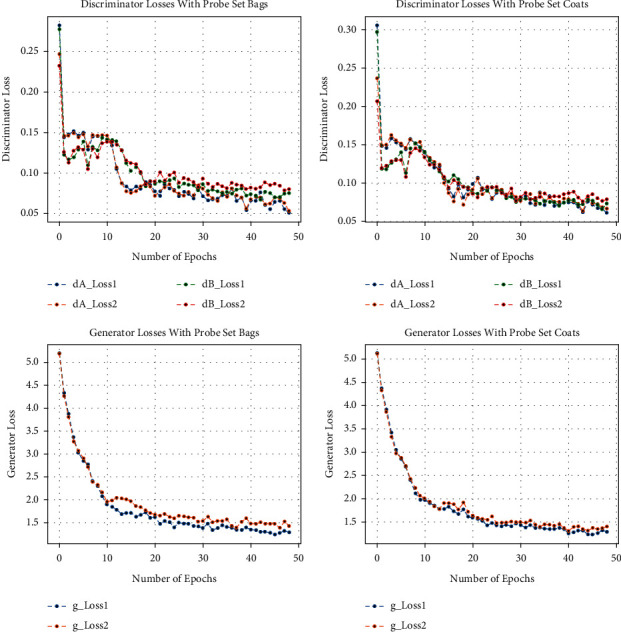
Loss of generators on number of steps per epoch with coats.

Besides this, at the time of training of CCGANs, loss values for generators and discriminators of both domains are shown in Figure 3. In Figure 3, the dA_loss1 is the loss of discriminator on real examples of domain A images, that is, bag or coat images, whereas dA_loss2 is the loss of discriminator of domain A on fake examples, i.e., artificially generated bags or coats images. Similarly, dB_loss1 and dB_loss2 are the losses of discriminators of domain B on real and fake examples, i.e., original and translated normal GEIs. Moreover, the loss of generators of both domains is also given as g_loss1 and g_loss2 in Figure 3. Each of these losses is a weighted average of cycle consistency loss *L*_cyc_ (both forward and backward) and adversarial losses *L*_GAN_ as given in equation (10). All these losses are recorded as per epochs. Furthermore, we have also calculated the loss per example during training termed as the number of steps per epoch. The per example loss graphs for both of the probe sets is also given in Figures 4, 5, 6, and 7. In these figures, the *x*-axis represents the steps per epoch, and the *y*-axis denotes losses of discriminators and generators. We terminated training after 12400 steps (50 epochs) because there was no progress in losses beyond that.  Table 3 also includes a comparison with prior approaches that take strict covariate conditions into account. From Table 3, it is observed that our proposed approach outperforms the existing approaches.

## 5. Conclusion

Gait Recognition without human involvement is a complex task in computer vision because all deep learning algorithms are not as much better to capture every possible dynamic walk environment. Generally, the computer vision algorithms are trained on a particular type of data to extract unique features and then conduct classification based on those extracted features. However, in the case of gait recognition without human involvement, the covariate factors change the gait features of individuals present in GEIs from those on which the deep learning model was trained. These covariate factors are unknown to the model, causing it to perform badly. So, this research work uses CCGANs to reconstruct the GEIs in which the same features are recovered on which model is trained. The CCGAN model is trained in an unsupervised manner to find a corresponding normal GEI for the bag/coat GEI of a particular individual. Then, the resultant translated images are tested by the CNN model. Moreover, we have achieved a very encouraging accuracy score of 79% in carrying conditions and also acceptable performance in clothing conditions. This work is further improved and extended using various sensors, to acquire gait data instead of only visual data and combine it with deep learning techniques to make the system more accurate and reliable.

## Figures and Tables

**Table 1:** Notations and definitions.

Notations	Definitions
**D**_**A**_	Discriminator model of domain A (probe bags/coats)
**D**_**B**_	Discriminator model of domain B (normal GEIs)
**A**	Training samples of domain A {*a*_*i*_}_*i*=1_^*N*^ ∈ *A*
**B**	Training samples of domain B {*b*_*i*_}_*i*=1_^*N*^ ∈ *B*
**F**{(**b**)}	Translated normal GEIs
**L**_**c** **y** **c**_	Cycle consistency loss function
**L**_**G** **A** **N**_	Adversarial loss function
**G**	Translator of domain A
**F**	Translator of domain B
**m** **i** **n**	Minimizing the variable
**m** **a** **x**	Maximizing the variable

**Table 2:** Results of CNN and CCGANs.

Sr. no.	Probe set	Accuracy (%)	Precision	Recall	F1score
1	Normal (CNN)	97.98	0.98	0.97	0.97
2	Bags (CNN)	38	0.29	0.38	0.31
3	Coats (CNN)	24.19	0.17	0.24	0.19
5	Bags (CNN + CCGANs)	**79**	**0.72**	**0.79**	**0.74**
6	Coats (CNN + CCGANs)	**51.8**	**0.38**	**0.51**	**0.41**

**Table 3:** Comparative analysis with the existing work under covariate conditions.

Sr. no.	Method	Normal	Bags	Coats (%)	Average accuracy (%)	Covariate
1	Bashir et al. [13]	100.0%	78.3%	44.4	74.2	Yes
2	Gupta et al. [18]	NA	86.2%	61.4	73.8	Yes
3	Hawas et al. [23]	97.6%	45.3%	49.6	64.1	Yes
4	Yu et al. [50]	95.97%	65.32%	42.74	68.01	Yes
5	Yao et al. [27]	NA	NA	38	38	Yes
6	Su et al. [25]	93.2%	72.8%	59.1	75.03	Yes
7	Proposed method	**97.98%**	**79%**	**51.8**	**76.26**	**Yes**

## Data Availability

The data sets generated during and analyzed during the current study are available from the corresponding author upon reasonable request.
